# Ethyl 2-(4-nitro­phen­oxy)acetate

**DOI:** 10.1107/S1600536812017242

**Published:** 2012-04-25

**Authors:** Su-Wen Sun

**Affiliations:** a College of Chemistry and Chemical Engineering, Southeast University, Nanjing 210096, People’s Republic of China

## Abstract

In the title mol­ecule, C_10_H_11_NO_5_, the methyl C atom deviates by 0.830 (6) Å from the mean plane of the remaining non-H atoms. In the crystal, weak C—H⋯O hydrogen bonds link the mol­ecules into layers parallel to the *bc* plane.

## Related literature

For the structure of *tert*-butyl 2-(4-nitro­phen­oxy)acetate, see: Ali *et al.* (2011 ▶). For general background to ferroelectric organics, see: Fu *et al.* (2009 ▶); Ye *et al.* (2006 ▶).
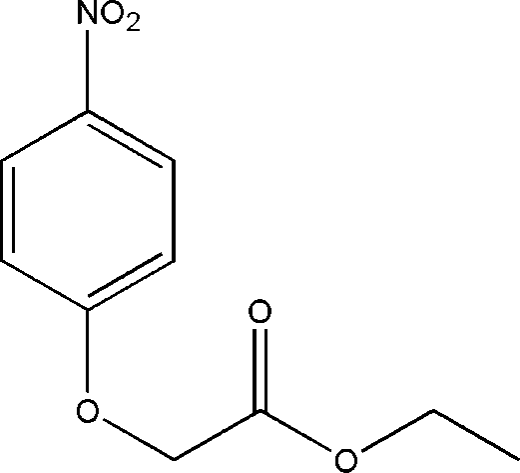


## Experimental

### 

#### Crystal data

C_10_H_11_NO_5_
*M* *_r_* = 225.20
Monoclinic, 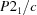

*a* = 5.3848 (11) Å
*b* = 8.4482 (17) Å
*c* = 24.238 (5) Å
β = 92.59 (3)°
*V* = 1101.5 (4) Å^3^
*Z* = 4
Mo *K*α radiation
μ = 0.11 mm^−1^
*T* = 293 K
0.3 × 0.3 × 0.2 mm

#### Data collection

Rigaku Mercury CCD diffractometer
Absorption correction: multi-scan (*CrystalClear*; Rigaku, 2005 ▶) *T*_min_ = 0.968, *T*_max_ = 0.978
9279 measured reflections
2169 independent reflections
1157 reflections with *I* > 2σ(*I*)
*R*_int_ = 0.089

#### Refinement

*R*[*F* ^2^ > 2σ(*F* ^2^)] = 0.068
*wR*(*F* ^2^) = 0.156
*S* = 1.01
2169 reflections
146 parameters
H-atom parameters constrained
Δρ_max_ = 0.20 e Å^−3^
Δρ_min_ = −0.21 e Å^−3^

### 

Data collection: *CrystalClear* (Rigaku, 2005 ▶); cell refinement: *CrystalClear*; data reduction: *CrystalClear*; program(s) used to solve structure: *SHELXS97* (Sheldrick, 2008 ▶); program(s) used to refine structure: *SHELXL97* (Sheldrick, 2008 ▶); molecular graphics: *SHELXTL* (Sheldrick, 2008 ▶); software used to prepare material for publication: *SHELXL97*.

## Supplementary Material

Crystal structure: contains datablock(s) I, global. DOI: 10.1107/S1600536812017242/cv5259sup1.cif

Structure factors: contains datablock(s) I. DOI: 10.1107/S1600536812017242/cv5259Isup2.hkl

Supplementary material file. DOI: 10.1107/S1600536812017242/cv5259Isup3.cml

Additional supplementary materials:  crystallographic information; 3D view; checkCIF report

## Figures and Tables

**Table 1:** Hydrogen-bond geometry (Å, °)

*D*—H⋯*A*	*D*—H	H⋯*A*	*D*⋯*A*	*D*—H⋯*A*
C2—H2*A*⋯O4^i^	0.93	2.53	3.177 (4)	127
C10—H10*B*⋯O1^ii^	0.96	2.58	3.513 (6)	164

Symmetry codes: (i) 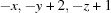; (ii) 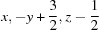.

**Fig. 1.**
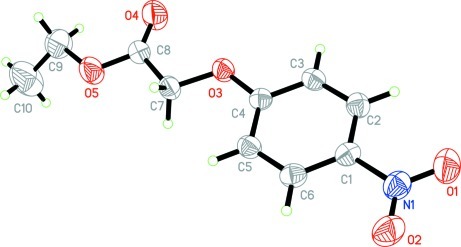
The molecular structure of (I) showing the atomic numbering and 40% probability displacement ellipsoids.

## supplementary crystallographic information

### Comment

The title compound, (I), has been obtain during the search for new organic compounds which demonstrate ferroelectric phase changes (Fu *et al.*, 2009; Ye *et al.*, 2006). Though the measured dielectric constant of (I) showed no dielectric disuniformity in the range 120–385 K (mp.393–402 K), herewith we present its crystal structure.

In (I) (Fig. 1), all bond lengths and angles are normal and correspond to those observed in *tert*-butyl 2-(4-nitrophenoxy)acetate (Ali *et al.*, 2011). Atom C10 deviates at 0.830 (6) Å from the mean plane of the rest non-H atoms.Weak intermolecular C—H···O hydrogen bonds (Table 1) link the molecules into layers parallel to *bc* plane.

### Experimental

4-Nitro-phenol (7 g) and 1-Bromo-butan-2-one (8.5 g) were dissolved in acetone, kalium carbonicum (7.5 g) was added to the mixture. Then the mixture was heated and refluxed by mechanical stirring at 70°C. Yellow solid was filtered off. This solid was dissolved in ethanol and single crystals suitable for X-ray structure analysis were obtained by the slow evaporation of the above solution after 3 days in air.

### Refinement

All H atoms were placed in calculated positions (N—H = 0.89 Å; C—H = 0.93 Å for C*sp^2^* atoms and C—H = 0.96 Å and 0.97 Å for C*sp^3^* atoms), assigned fixed *U*_iso_ values [*U*_iso_ = 1.2*U*eq(C*sp^2^*) and 1.5*U*eq(C*sp^3^*,*N*)] and allowed to ride.

### Crystal data

**Table:**

C_10_H_11_NO_5_	*F*(000) = 472
*M**_r_* = 225.20	*D*_x_ = 1.358 Mg m^−^^3^
Monoclinic, *P*2_1_/*c*	Mo *K*α radiation, λ = 0.71073 Å
Hall symbol: -P 2ybc	Cell parameters from 3450 reflections
*a* = 5.3848 (11) Å	θ = 3.4–26.0°
*b* = 8.4482 (17) Å	µ = 0.11 mm^−^^1^
*c* = 24.238 (5) Å	*T* = 293 K
β = 92.59 (3)°	Block, colourless
*V* = 1101.5 (4) Å^3^	0.3 × 0.3 × 0.2 mm
*Z* = 4	

### Data collection

**Table:**

Rigaku Mercury CCD diffractometer	2169 independent reflections
Radiation source: fine-focus sealed tube	1157 reflections with *I* > 2σ(*I*)
Graphite monochromator	*R*_int_ = 0.089
ω scans	θ_max_ = 26.0°, θ_min_ = 3.4°
Absorption correction: multi-scan (*CrystalClear*; Rigaku, 2005)	*h* = −6→6
*T*_min_ = 0.968, *T*_max_ = 0.978	*k* = −10→10
9279 measured reflections	*l* = −29→29

### Refinement

**Table:**

Refinement on *F*^2^	Secondary atom site location: difference Fourier map
Least-squares matrix: full	Hydrogen site location: inferred from neighbouring sites
*R*[*F*^2^ > 2σ(*F*^2^)] = 0.068	H-atom parameters constrained
*wR*(*F*^2^) = 0.156	*w* = 1/[σ^2^(*F*_o_^2^) + (0.05*P*)^2^ + 0.35*P*] where *P* = (*F*_o_^2^ + 2*F*_c_^2^)/3
*S* = 1.01	(Δ/σ)_max_ < 0.001
2169 reflections	Δρ_max_ = 0.20 e Å^−^^3^
146 parameters	Δρ_min_ = −0.21 e Å^−^^3^
0 restraints	Extinction correction: *SHELXL97* (Sheldrick, 2008), Fc^*^=kFc[1+0.001xFc^2^λ^3^/sin(2θ)]^-1/4^
Primary atom site location: structure-invariant direct methods	Extinction coefficient: 0.011 (2)

### Special details

**Table:**

Geometry. All e.s.d.'s (except the e.s.d. in the dihedral angle between two l.s. planes) are estimated using the full covariance matrix. The cell e.s.d.'s are taken into account individually in the estimation of e.s.d.'s in distances, angles and torsion angles; correlations between e.s.d.'s in cell parameters are only used when they are defined by crystal symmetry. An approximate (isotropic) treatment of cell e.s.d.'s is used for estimating e.s.d.'s involving l.s. planes.
Refinement. Refinement of *F*^2^ against ALL reflections. The weighted *R*-factor *wR* and goodness of fit *S* are based on *F*^2^, conventional *R*-factors *R* are based on *F*, with *F* set to zero for negative *F*^2^. The threshold expression of *F*^2^ > σ(*F*^2^) is used only for calculating *R*-factors(gt) *etc*. and is not relevant to the choice of reflections for refinement. *R*-factors based on *F*^2^ are statistically about twice as large as those based on *F*, and *R*- factors based on ALL data will be even larger.

### Fractional atomic coordinates and isotropic or equivalent isotropic displacement parameters (Å^2^)

**Table:**

	*x*	*y*	*z*	*U*_iso_*/*U*_eq_	
O3	0.2402 (3)	0.9062 (2)	0.43476 (7)	0.0593 (5)	
C4	0.3764 (4)	0.8226 (3)	0.47348 (10)	0.0502 (6)	
C3	0.2863 (5)	0.8248 (3)	0.52630 (10)	0.0559 (7)	
H3A	0.1438	0.8824	0.5331	0.067*	
C1	0.6184 (5)	0.6596 (3)	0.55727 (11)	0.0540 (7)	
C7	0.3224 (5)	0.9043 (3)	0.38008 (10)	0.0598 (7)	
H7A	0.3228	0.7968	0.3661	0.072*	
H7B	0.4902	0.9458	0.3794	0.072*	
C6	0.7118 (5)	0.6582 (3)	0.50557 (11)	0.0600 (7)	
H6A	0.8565	0.6024	0.4992	0.072*	
O5	0.2362 (4)	1.0156 (3)	0.29508 (8)	0.0930 (8)	
C5	0.5909 (4)	0.7396 (3)	0.46316 (11)	0.0568 (7)	
H5A	0.6527	0.7389	0.4279	0.068*	
N1	0.7460 (5)	0.5687 (3)	0.60109 (11)	0.0713 (7)	
C2	0.4062 (5)	0.7424 (3)	0.56848 (11)	0.0588 (7)	
H2A	0.3454	0.7424	0.6038	0.071*	
O1	0.6650 (5)	0.5737 (3)	0.64727 (10)	0.1141 (10)	
O2	0.9260 (4)	0.4885 (3)	0.59049 (9)	0.0938 (8)	
C8	0.1488 (5)	1.0048 (4)	0.34495 (11)	0.0640 (8)	
O4	−0.0361 (4)	1.0635 (3)	0.35870 (9)	0.1103 (10)	
C9	0.0983 (7)	1.1139 (6)	0.25442 (15)	0.1140 (14)	
H9A	−0.0259	1.0504	0.2344	0.137*	
H9B	0.0140	1.1987	0.2730	0.137*	
C10	0.2659 (8)	1.1779 (7)	0.21723 (18)	0.157 (2)	
H10A	0.1762	1.2415	0.1902	0.235*	
H10B	0.3491	1.0935	0.1991	0.235*	
H10C	0.3863	1.2424	0.2371	0.235*	

### Atomic displacement parameters (Å^2^)

**Table:**

	*U*^11^	*U*^22^	*U*^33^	*U*^12^	*U*^13^	*U*^23^
O3	0.0581 (11)	0.0663 (12)	0.0541 (11)	0.0116 (9)	0.0075 (8)	0.0024 (9)
C4	0.0496 (15)	0.0462 (15)	0.0547 (15)	−0.0030 (12)	0.0012 (12)	−0.0033 (13)
C3	0.0522 (15)	0.0567 (17)	0.0594 (16)	−0.0004 (13)	0.0083 (12)	−0.0025 (14)
C1	0.0535 (16)	0.0467 (15)	0.0612 (17)	−0.0028 (13)	−0.0046 (13)	0.0025 (14)
C7	0.0612 (16)	0.0655 (18)	0.0534 (16)	0.0049 (14)	0.0104 (13)	0.0001 (14)
C6	0.0551 (16)	0.0554 (17)	0.0694 (18)	0.0060 (13)	0.0020 (14)	−0.0043 (16)
O5	0.0837 (14)	0.130 (2)	0.0668 (13)	0.0279 (14)	0.0152 (11)	0.0294 (14)
C5	0.0539 (16)	0.0596 (17)	0.0574 (15)	0.0025 (13)	0.0079 (12)	−0.0056 (14)
N1	0.0725 (17)	0.0681 (17)	0.0725 (18)	−0.0037 (14)	−0.0054 (14)	0.0084 (15)
C2	0.0636 (17)	0.0562 (17)	0.0568 (16)	−0.0076 (14)	0.0050 (13)	0.0006 (14)
O1	0.1161 (19)	0.153 (3)	0.0734 (16)	0.0334 (17)	0.0087 (14)	0.0313 (17)
O2	0.0892 (16)	0.0956 (17)	0.0954 (17)	0.0293 (14)	−0.0086 (13)	0.0132 (14)
C8	0.0609 (17)	0.076 (2)	0.0554 (16)	0.0006 (16)	0.0055 (14)	0.0000 (15)
O4	0.0937 (17)	0.161 (3)	0.0771 (15)	0.0615 (17)	0.0125 (13)	0.0127 (15)
C9	0.101 (3)	0.160 (4)	0.080 (2)	0.023 (3)	−0.002 (2)	0.043 (3)
C10	0.149 (4)	0.196 (5)	0.129 (4)	0.053 (4)	0.043 (3)	0.081 (4)

### Geometric parameters (Å, º)

**Table:**

O3—C4	1.362 (3)	O5—C8	1.320 (3)
O3—C7	1.416 (3)	O5—C9	1.465 (4)
C4—C5	1.384 (3)	C5—H5A	0.9300
C4—C3	1.390 (3)	N1—O2	1.220 (3)
C3—C2	1.374 (3)	N1—O1	1.220 (3)
C3—H3A	0.9300	C2—H2A	0.9300
C1—C6	1.371 (3)	C8—O4	1.174 (3)
C1—C2	1.377 (3)	C9—C10	1.412 (5)
C1—N1	1.458 (3)	C9—H9A	0.9700
C7—C8	1.499 (4)	C9—H9B	0.9700
C7—H7A	0.9700	C10—H10A	0.9600
C7—H7B	0.9700	C10—H10B	0.9600
C6—C5	1.376 (3)	C10—H10C	0.9600
C6—H6A	0.9300		
			
C4—O3—C7	117.25 (19)	C4—C5—H5A	120.4
O3—C4—C5	124.5 (2)	O2—N1—O1	122.2 (3)
O3—C4—C3	115.4 (2)	O2—N1—C1	119.5 (3)
C5—C4—C3	120.1 (2)	O1—N1—C1	118.3 (3)
C2—C3—C4	120.5 (2)	C3—C2—C1	118.5 (2)
C2—C3—H3A	119.8	C3—C2—H2A	120.8
C4—C3—H3A	119.8	C1—C2—H2A	120.8
C6—C1—C2	121.8 (2)	O4—C8—O5	125.0 (3)
C6—C1—N1	118.8 (2)	O4—C8—C7	126.4 (3)
C2—C1—N1	119.5 (2)	O5—C8—C7	108.7 (2)
O3—C7—C8	108.2 (2)	C10—C9—O5	109.1 (3)
O3—C7—H7A	110.1	C10—C9—H9A	109.9
C8—C7—H7A	110.1	O5—C9—H9A	109.9
O3—C7—H7B	110.1	C10—C9—H9B	109.9
C8—C7—H7B	110.1	O5—C9—H9B	109.9
H7A—C7—H7B	108.4	H9A—C9—H9B	108.3
C1—C6—C5	119.8 (2)	C9—C10—H10A	109.5
C1—C6—H6A	120.1	C9—C10—H10B	109.5
C5—C6—H6A	120.1	H10A—C10—H10B	109.5
C8—O5—C9	117.7 (3)	C9—C10—H10C	109.5
C6—C5—C4	119.3 (2)	H10A—C10—H10C	109.5
C6—C5—H5A	120.4	H10B—C10—H10C	109.5

### Hydrogen-bond geometry (Å, º)

**Table:**

*D*—H···*A*	*D*—H	H···*A*	*D*···*A*	*D*—H···*A*
C2—H2*A*···O4^i^	0.93	2.53	3.177 (4)	127
C10—H10*B*···O1^ii^	0.96	2.58	3.513 (6)	164

Symmetry codes: (i) −*x*, −*y*+2, −*z*+1; (ii) *x*, −*y*+3/2, *z*−1/2.
